# Cyclobutane dication, (CH_2_)_4_^2+^: a model for a two-electron four-center (2e-4c) Woodward–Hoffmann frozen transition state

**DOI:** 10.3762/bjoc.15.148

**Published:** 2019-07-03

**Authors:** G K Surya Prakash, Golam Rasul

**Affiliations:** 1 Loker Hydrocarbon Research Institute and Department of Chemistry, University of Southern California, University Park, Los Angeles, CA 90089-1661, USA

**Keywords:** cyclobutane dication, 2e-4c bond, frozen transition state, Woodward–Hoffmann rule

## Abstract

The structures of the elusive cyclobutane dication, (CH_2_)_4_^2+^, were investigated at the MP2/cc-pVTZ and CCSD(T)/cc-pVTZ levels. Calculations show that the two-electron four-center (2e-4c) bonded structure **1** involving four carbon atoms is a minimum. The structure contains formally two tetracoordinate and two pentacoordinate carbons. The non-classical σ-delocalized structure can be considered as a prototype for a 2e-4c Woodward–Hoffmann frozen transition state. The planar rectangular shaped structure **2** with a 2e-4c bond was found not to be a minimum.

## Introduction

**Scheme 1:**
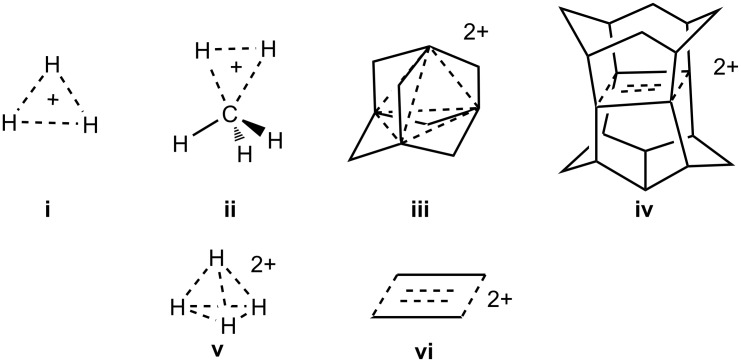
Examples of 2e-3c and 2e-4c bonded structures.

The protonated hydrogen cation (H_3_^+^, **i**) is the simplest known structure involving a two-electron three-center (2e-3c) bond (Scheme 1). The first spectroscopic detection of the H_3_^+^ ion was reported by Oka in 1980 [1]. Similarly, the structure of five coordinated protonated methane (CH_5_^+^, **ii**), a prototype of nonclassical carbocations, could not be interpreted by the classical tetravalency bonding concept [2–3] also requiring the engagement of a 2e-3c bond as suggested by Olah in 1969 [4–5]. A compelling number and huge array of carbocations involving higher coordinate carbon is by now realized by experimental studies.

Hypercarbon chemistry covers in addition to carbocations also carboranes, carbon-bridged organometallics, carbonyl clusters, along with others. The rapidly evolving field has been extensively surveyed [6]. In comparison, structures of the carbocations involving a two-electron four-center (2e-4c) bond are rare. This type of bonding could occur in rigid frameworks such as in 1,3-dehydro-5,7-adamantanediyl dication (**iii**) [7] and pagodane dication (**iv**) [8]. The question is can a 2e-4c bond exist in a molecular structure involving four atoms without any rigid frameworks such as in the diprotonated hydrogen (H_4_^2+^, **v**) and the cyclobutane dication (CH_2_)_4_^2+^ (**vi**)?

The structures of H_4_^2+^ (including **v**) were previously analyzed at various theoretical levels including CISD/6-311++G(d,2pd) by von Ragué Schleyer, Koch and co-workers [9]. No structure with four connected hydrogens, however, was found to be a minimum on the potential energy surface (PES) of H_4_^2+.^ The structure of the rectangular shaped cyclobutane dication (CH_2_)_4_^2+^ (**vi**) was computed by Olah, Prakash et al. [8,10] using semiempirical and ab initio methods and later by Herges, von Ragué Schleyer, Schindler and Fessner [11] using an ab initio method. The various levels of calculations including MP2/6-31G* indicated that the structure **vi** was not a minimum [11]. The 1,3-dehydro-5,7-adamantanediyl dication (**iii**) [7] with 2e-4c bonding was generated and identified by ^13^C NMR spectroscopic and theoretical methods corresponding to a three dimensional aromaticity. The four bridge-head p-orbitals overlap inward in a tetrahedral fashion involving two electrons.

We have now extended our study to obtain information on the structure, stabilities and possible rearrangement pathways of the elusive cyclobutane dication. The species is an example of the simplest carbodication containing a 2e-4c bond and can be considered as a prototype for a frozen Woodward–Hoffmann transition state analog [12–13]. The 2e-4c delocalized σ-bishomoaromatic system is representative of a 2e-aromatic pericycliclic species. This type of system may be considered as the transition state of the allowed cycloaddition of ethylene to ethylene dication. Electron delocalizations take place in the plane of the conjugated systems unlike cyclobutadiene dication (**vi**), where delocalization takes place through conventional p-type orbitals. GIAO-CCSD(T) derived ^13^C NMR chemical shifts of the structures were also computed to probe the nature and extent of the positive charge delocalization.

## Calculations

The Gaussian 09 program [14] was employed for geometry optimizations and frequency calculations. Vibrational frequencies at the MP2/cc-pVTZ//MP2/cc-pVTZ level were used to characterize stationary points as minima (NIMAG (number of imaginary frequencies) = 0 or transition state NIMAG = 1) and to compute zero point vibrational energies (ZPE), which were scaled by a factor of 0.96 [15]. CCSD(T)/cc-pVTZ optimizations and GIAO-CCSD(T) ^13^C NMR chemical shifts calculations by the GIAO (Gauge Invariant Atomic Orbitals) method [16–19] using tzp and qzp basis sets, which were optimized by Schäfer, Horn and Ahlrichs [20–21], have been performed with the CFOUR program [22–23]. The ^13^C NMR chemical shifts were computed using TMS (calculated absolute shift, i.e., σ(C) = 197.9 (GIAO-CCSD(T)) as a reference.

## Results and Discussion

**Figure 1:**
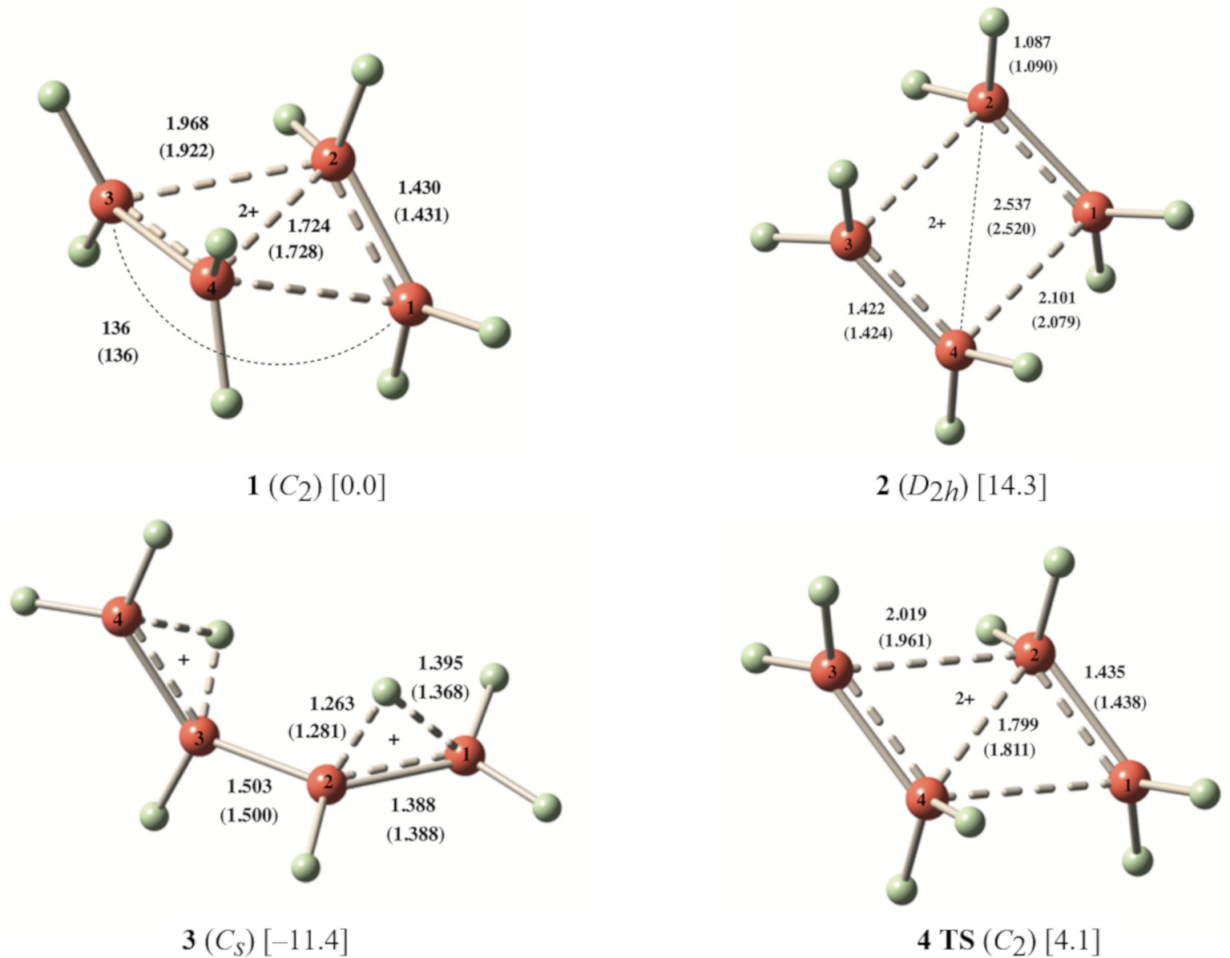
CCSD(T)/cc-pVTZ (MP2/cc-pVTZ) optimized structures and relative energies [in kcal/mol] of **1**–**4**.

**Figure 2:**
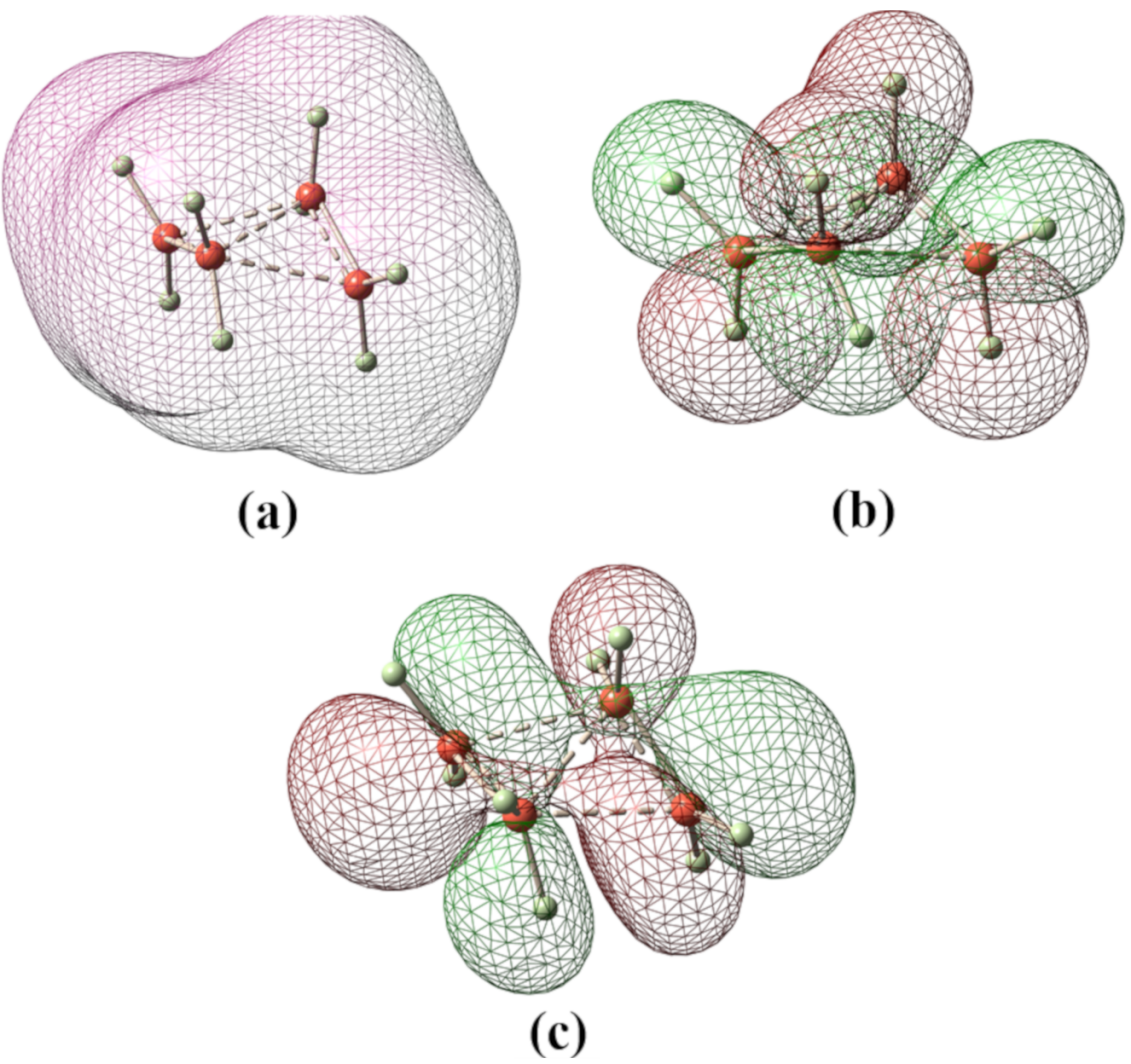
(a) Total electron density, (b) HOMO and (c) LUMO of the dication **1**; coefficients are calculated at the HF/6-31G* level.

Structures of **1–4** were optimized at the MP2/cc-pVTZ and CCSD(T)/cc-PVTZ levels. CCSD(T)/cc-PVTZ structures will be discussed throughout unless otherwise stated. Dication **1** is expected to form by removal of two electrons from cyclobutane. At both MP2/cc-pVTZ and CCSD(T)/cc-PVTZ levels, the *C*_2_ symmetric form **1** (Figure 1) was found to be a minimum for (CH_2_)_4_^2+^. This is confirmed by frequency calculations at the corresponding levels. The ring of the structure **1** embraces a puckered conformation with a puckering angle (the angle between the two three-membered rings) of 136° as shown in Figure 1. The structure contains conventionally two tetracoordinate and two pentacoordinate carbons. It resembles a complex between two ethylene radical cations, (CH_2_)_4_^2+^, culminating in the formation of a 2e-4c bond. Total electron density, HOMO and LUMO of the dication **1** are depicted in Figure 2. In such a small ring doubly charged system, charge–charge repulsion is certainly strong, but the bonding interactions as well as charge delocalization are good enough to counter this repulsion. The C1–C4 and C2–C3 bonds (1.968 Å) were calculated to be significantly longer than the C1–C2 and C3–C4 bonds (1.430 Å). The *D*_2_*_h_* symmetric structure **2** was also computed for comparison with the structure **1**. Computed vibrational frequencies at the MP2/cc-pVTZ//MP2/cc-pVTZ level indicated that the rectangular shaped structure **2** could not be a minimum as it contains two imaginary frequencies (NIMAG = 2, corresponding to the vibrations of the two ethylene units in plane and out of plane).

Moreover, structure **2** was also found to be notably disfavored over the structure **1** by 14.3 kcal/mol at the CCSD(T)/cc-PVTZ + ZPE level (Figure 1, Table S9 in Supporting Information File 1). Attempts to find a minimum for the open 1,4-butanediyl dication, bearing two primary carbenium centers failed due to automatic transformation to the thermodynamically more stable hydrogen-bridged structure **3** (Figure 1).

The twisted angle between the planes of the hydrogen-bridged units in **3** was found to be 91.4°. Expectedly, the structure **3** was found to be favored over the structure **1** by 11.4 kcal/mol (Figure 1, Table S9 in Supporting Information File 1). Planar parallelogram-shaped structure **4** was identified as the transition state for the transformation of **1** to **3** (Figure 1). The structure **4** lies 4.1 kcal/mol above **1**. Dissociation of **1** leading to two ethylene radical cations (CH_2_=CH_2_^·+^) was also considered. The process was found to be exothermic by 12.8 kcal/mol at the CCSD(T)/cc-PVTZ + ZPE level. We tried, but could not locate a transition state for the dissociation process at the CCSD(T)/cc-PVTZ level.

The ^13^C NMR chemical shifts of the structures **1**–**3** were computed by employing GIAO-MP2 and GIAO-CCSD(T) methods using CCSD(T)/cc-pVTZ geometries. GIAO-CCSD(T) calculated ^13^C NMR chemical shifts for **1** show that the C1 and C2 carbons are deshielded at δ 216.6 and 40.2 ppm, respectively, indicating a nonclassical nature of the ion in accord with its tetra- and pentacoordinate nature. Computed ^13^C NMR chemical shifts of the structure **1** are given in Table 1. Vibrational frequencies of the structure **1** are given in Table S10 (see Supporting Information File 1).

**Table 1:** GIAO calculated^a 13^C NMR chemical shifts using CCSD(T)/cc-pVTZ geometries.

no	atom	GIAO-MP2/tzp	GIAO-CCSD(T)/tzp

**1**	C1, C3	217.4	216.6
	C2, C4	39.9	40.2
**2**	C1, C2, C3, C4	196.6	195.8
**3**	C1, C4	182.0	179.5
	C2, C3	134.4	132.1

^a^The ^13^C NMR chemical shifts were referenced to TMS, for numbering scheme please see Figure 1.

## Conclusion

The present study at the MP2/cc-pVTZ and CCSD(T)/cc-pVTZ levels shows that the cyclobutane dication, (CH_2_)_4_^2+^ (**1**), a prototype for a 2e-4c Woodward–Hoffmann frozen transition state, is a viable minimum on its potential energy surface. The structure contains formally two tetracoordinate and two pentacoordinate carbons. It resembles a complex between two ethylene radical ions, (C_2_H_4_)^2·+^, culminating in the formation of a 2e-4c bond involving four carbon atoms. The planar rectangular shaped structure **2** with a 2e-4c bond was found to be not a minimum.

## Supporting Information

File 1 MP2/cc-pVTZ and CCSD(T)/cc-pVTZ optimized Cartesian coordinates of **1**–**4**, energies, ZPE and relative energies of **1**–**4** (Table S9) and calculated frequencies and IR intensities of **1** (Table S10).
