# Supplementary material for: Cyclobutane dication, (CH2)42+: a model for a two-electron four-center (2e-4c) Woodward–Hoffmann frozen transition state
Source: Beilstein J Org Chem. 2019 Jul 3;15:1475–9. doi: 10.3762/bjoc.15.148 (PMC6633612; doi:10.3762/bjoc.15.148)
Supplement: File 1 — MP2/cc-pVTZ and CCSD(T)/cc-pVTZ optimized Cartesian coordinates of 1–4, energies, ZPE and relative energies of 1–4 (Table S9) and calculated frequencies and IR intensities of 1 (Table S10). [file Beilstein_J_Org_Chem-15-1475-s001.pdf]

BEILSTEIN JOURNAL OF ORGANIC CHEMISTRY

Supporting Information

for

Cyclobutane dication, $(CH_2)_4^{2+}$: a model for a two-electron four-center (2e-4c) Woodward–Hoffmann frozen transition state

G. K. Surya Prakash and Golam Rasul

Beilstein J. Org. Chem. **2019,** *15,* 1475–1479. doi:10.3762/bjoc.15.148

MP2/cc-pVTZ and CCSD(T)/cc-pVTZ optimized Cartesian coordinates of 1–4, energies, ZPE and relative energies of 1–4 (Table S9) and calculated frequencies and IR intensities of 1 (Table S10)

License and Terms: This is a supporting information file under the terms of the Creative Commons Attribution License (http://creativecommons.org/licenses/by/4.0). Please note that the reuse, redistribution and reproduction in particular requires that the authors and source are credited.
The license is subject to the *Beilstein Journal of Organic Chemistry* terms and conditions: (https://www.beilstein-journals.org/bjoc)

Table of Contents

Table S1. MP2/cc-pVTZ optimized Cartesian coordinates of **1**

Table S2. MP2/cc-pVTZ optimized Cartesian coordinates of **2**

Table S3. MP2/cc-pVTZ optimized Cartesian coordinates of **3**

Table S4. MP2/cc-pVTZ optimized Cartesian coordinates of **4 TS**

Table S5. CCSD(T)/cc-pVTZ optimized Cartesian coordinates of **1**

Table S6. CCSD(T)/cc-pVTZ optimized Cartesian coordinates of **2**

Table S7. CCSD(T)/cc-pVTZ optimized Cartesian coordinates of **3**

Table S8. CCSD(T)/cc-pVTZ optimized Cartesian coordinates of **4 TS**

Table S9. Energies, ZPE and relative energies of **1** – **4**

Table S10. Calculated frequencies and IR intensities of **1**

Table S1. MP2/cc-pVTZ optimized Cartesian coordinates of **1**

C	0.5927	0.6283	-0.2817
C	-0.5927	-0.6283	-0.2817
C	-0.5927	1.2138	0.2673
C	0.5927	-1.2138	0.2673
H	0.8708	0.7741	-1.3207
H	-0.8708	-0.7741	-1.3207
H	1.4318	0.5075	0.4403
H	-1.4318	-0.5075	0.4403
H	-1.2920	1.7460	-0.3780
H	1.2920	-1.7460	-0.3780
H	-0.7455	1.2479	1.3448
H	0.7455	-1.2479	1.3448

Table S2. MP2/cc-pVTZ optimized Cartesian coordinates of **2**

C	-1.0393	0.7122	0.0000
C	-1.0393	-0.7122	0.0000
C	1.0393	0.7122	0.0000
C	1.0393	-0.7122	0.0000
H	-1.1500	1.2527	0.9395
H	-1.1500	-1.2527	0.9395
H	-1.1500	1.2527	-0.9395
H	-1.1500	-1.2527	-0.9395
H	1.1500	1.2527	-0.9395
H	1.1500	-1.2527	-0.9395
H	1.1500	1.2527	0.9395
H	1.1500	-1.2527	0.9395

Table S3. MP2/cc-pVTZ optimized Cartesian coordinates of **3**

C	-0.2256	0.7154	0.4570
C	0.2256	-0.7154	0.4570
C	0.2256	1.7456	-0.3556
C	-0.2256	-1.7456	-0.3556
H	-0.8516	0.9691	1.3156
H	0.8516	-0.9691	1.3156
H	-0.1297	2.7574	-0.1674
H	0.1297	-2.7574	-0.1674

H	0.8803	1.5714	-1.2060
H	-0.8803	-1.5714	-1.2060
H	-0.9393	1.0552	-0.5511
H	0.9393	-1.0552	-0.5511

Table S4. MP2/cc-pVTZ optimized Cartesian coordinates of **4 TS**

C	0.6356	0.6444	-0.0003
C	-0.6356	-0.6444	-0.0003
C	-0.6356	1.3160	0.0003
C	0.6356	-1.3160	0.0003
H	1.1769	0.6676	-0.9480
H	-1.1769	-0.6676	-0.9480
H	1.1776	0.6673	0.9470
H	-1.1776	-0.6673	0.9470
H	-1.0948	1.6304	-0.9351
H	1.0948	-1.6304	-0.9351
H	-1.0939	1.6304	0.9362
H	1.0939	-1.6304	0.9362

Table S5. CCSD(T)/cc-pVTZ optimized Cartesian coordinates of **1**

C	0.5814	0.6366	-0.2881
C	-0.5814	-0.6366	-0.2881
C	-0.5814	1.2486	0.2752
C	0.5814	-1.2486	0.2752
H	0.8557	0.7932	-1.3241
H	-0.8557	-0.7932	-1.3241
H	1.4297	0.5091	0.4150
H	-1.4297	-0.5091	0.4150
H	-1.2874	1.7740	-0.3645
H	1.2874	-1.7740	-0.3645
H	-0.7348	1.2685	1.3506
H	0.7348	-1.2685	1.3506

Table S6. CCSD(T)/cc-pVTZ optimized Cartesian coordinates of **2**

C	-1.0507	0.7111	0.0000
C	-1.0507	-0.7111	0.0000
C	1.0507	0.7111	0.0000
C	1.0507	-0.7111	0.0000
H	-1.1568	1.2514	0.9371

H	-1.1568	-1.2514	0.9371
H	-1.1568	1.2514	-0.9371
H	-1.1568	-1.2514	-0.9371
H	1.1568	1.2514	-0.9371
H	1.1568	-1.2514	-0.9371
H	1.1568	1.2514	0.9371
H	1.1568	-1.2514	0.9371

Table S7. CCSD(T)/cc-pVTZ optimized Cartesian coordinates of **3**

C	-0.2397	0.7120	0.4350
C	0.2397	-0.7120	0.4350
C	0.2397	1.7628	-0.3348
C	-0.2397	-1.7628	-0.3348
H	-0.8837	0.9464	1.2818
H	0.8837	-0.9464	1.2818
H	-0.1329	2.7659	-0.1442
H	0.1329	-2.7659	-0.1442
H	0.9225	1.6112	-1.1653
H	-0.9225	-1.6112	-1.1653
H	-0.9275	1.0377	-0.5732
H	0.9275	-1.0377	-0.5732

Table S8. CCSD(T)/cc-pVTZ optimized Cartesian coordinates of **4 TS**

C	0.6212	0.6506	0.0000
C	-0.6212	-0.6506	0.0000
C	-0.6212	1.3682	-0.0000
C	0.6212	-1.3682	-0.0000
H	1.1709	0.6631	-0.9393
H	-1.1709	-0.6631	-0.9393
H	1.1708	0.6632	0.9394
H	-1.1708	-0.6632	0.9394
H	-1.0819	1.6748	-0.9350
H	1.0819	-1.6748	-0.9350
H	-1.0820	1.6749	0.9349
H	1.0820	-1.6749	0.9349

Table S9. Energies (-au), ZPE and relative energies of **1** – **4**

No.	MP2/cc-pVTZ	ZPE	CCSD(T)/cc-pVTZ	rel. energy[a]
		(kcal/mol)		(kcal/mol)
**1**	155.96386	64.6	156.09920	0.0
**2**	155.93888	63.6	156.07487	14.3
**3**	155.97491	62.5	156.11407	-11.4
**4 TS**	155.95750	64.9	156.09311	4.1

[a]*at the* CCSD(T)/cc-pVTZ//CCSD(T)/cc-pVTZ + *ZPE level*

Table S10. Calculated frequencies (cm^{-1}) and IR intensities (km/mol) of **1**

MP2/cc-pVTZ//MP2/cc-pVTZ		CCSD(T)/cc-pVTZ//CCSD(T)//cc-pVTZ	
freq.	inten.	freq.	inten.
210	165	191	184
212	37	204	39
363	6	333	10
564	8	552	7
752	192	740	143
780	7	751	5
840	67	846	57
876	14	865	16
997	98	974	168
1000	1	997	3
1085	28	1090	33
1135	6	1143	3
1170	0	1178	0
1189	107	1187	104
1219	8	1223	6
1225	30	1228	32
1265	105	1262	91
1292	11	1304	12
1437	13	1448	15
1446	144	1459	134
1507	10	1511	15
1530	0	1535	0
2898	328	2898	266
2920	17	2918	14
3116	111	3106	105
3117	8	3107	8
3205	15	3179	18
3217	173	3190	121
3240	28	3209	26
3241	204	3210	208
